# Twin-to-twin transfusion syndrome complicated with in utero limb ischemia of the donor twin – a case report

**DOI:** 10.1186/s12884-022-04429-0

**Published:** 2022-02-04

**Authors:** Agata Majewska, Robert Brawura-Biskupski-Samaha, Szymon Kozłowski, Dorota Bomba-Opoń, Iwona Szymusik, Olga Płaza, Mirosław Wielgoś

**Affiliations:** 1 1st Department of Obstetrics and Gynecology, Medical University of Warsaw, Warsaw, Poland; 2 The Institute of Mother and Child, Warsaw, Poland; 3 Students Scientific Association at the 1st Department of Obstetrics and Gynecology, Medical University of Warsaw, Warsaw, Poland

**Keywords:** Selective laser photocoagulation, Prenatal limb ischemia, Twin-to-twin transfusion syndrome

## Abstract

**Background:**

In utero limb ischemia is a rare complication of the monochorionic twin pregnancies complicated with twin to twin transfusion syndrome (TTTS). The condition is more often seen in recipient twins. There are few theories of the pathogenesis including in utero venous thromboembolism, but the cause remains unclear. However, limb ischemia is thought to be unrelated with any prenatal intervention.

**Case Presentation:**

We present a case of a monochorionic twin pregnancy complicated with TTTS admitted to the Clinic for selective fetoscopic laser photocoagulation. The invasive procedure failed due to poor visibility. In the following weeks of pregnancy, amnioreduction procedures were performed. At 28 weeks of gestation due to twin anemia-polycythemia sequence diagnosis the patient was qualified for cesarean section. Postnatally, the donor twin was diagnosed with lower right limb ischemic necrosis. The extremity was amputated 2 days later with an uncomplicated recovery. After speculations of the potential pathogeneses it was suggested that the ischemic limb occurred as a complication of the main condition – TTTS.

**Conclusions:**

In literature, there have been no cases reported of TTTS stage I complicated with donor twin limb ischemia. The actual cause of the in utero limb ischemic necrosis in monochorionic twins remains unknown. Nevertheless, increased attention to the potential complication after failed invasive procedures or conservative treatment should be required.

## Background

Twin-to-twin transfusion syndrome (TTTS) is one of the complications of monochorionic twin pregnancies, occurring in approximately 10–15% [1, 2]. The classification of severity of the disease is based on the Quintero staging system [3, 4]. Untreated TTTS has a very high mortality rate and morbidity including neurologic and cardiovascular complications [2, 5, 6]. Although there are few treatment strategies for managing TTTS, selective laser photocoagulation (SLP) is thought to be the most cost-effective treatment [7, 8]. SLP improves perinatal outcomes by decreasing the risk of fetal demise and postnatal complications [9, 10].

One of the rare complications of TTTS is prenatal limb ischemia. In most cases, it affects the recipient twin [11]. There are few theories of the pathogenesis including in utero venous thromboembolism, but the cause remains unclear [12, 13]. However, limb ischemia is thought to be unrelated with any prenatal intervention [14].

## Case Presentation

A 28-year-old multiparous patient was admitted to a tertiary referral center, 1^st^ Department of Obstetrics and Gynecology, Medical University of Warsaw at 22 weeks and 0 days with Quintero stage I twin-to-twin transfusion syndrome. The recipient twin had the deepest vertical pocket of 10.3 cm, while the donor had anhydramnios. Doppler evidence of cardiac dysfunction was seen in both fetuses. After informed consent the patient underwent selective laser photocoagulation. A fetoscope was introduced into the recipient twin sac visualizing the intertwin membrane and the chorionic plate. Arteriovenous anastomoses were evaluated, but after the first coagulation procedure visibility had deteriorated significantly due to bleeding from the entry site. It was decided to stop the procedure due to safety reasons. The patient was monitored weekly after the procedure with ultrasound scans. Due to persistent polyhydramnios of the recipient twin, amnioreduction procedures were performed. The patient underwent four procedures of amnioreduction with an interval of 7 days (the amount of amniotic fluid withdrawn ranged from 1700 to 2500 ml). Noteworthy was the colour of amniotic fluid, that was not transparent but brown suggesting the high risk of bleeding while attempting more invasive procedures. No fetal anomaly was visualized on follow up ultrasound scans.

At 28 weeks and 6 days the patient was diagnosed with twin anemia-polycythemia sequence stage 1(TAPS). Due to the high risk of bleeding from the entry site the patient was disqualified from any invasive procedure as treatment of TAPS. The patient was admitted to the hospital and qualified for prenatal corticosteroids (Betamethasone). After the antenatal steroid therapy, the patient was qualified for a caesarean section.

**Fig. 1:**
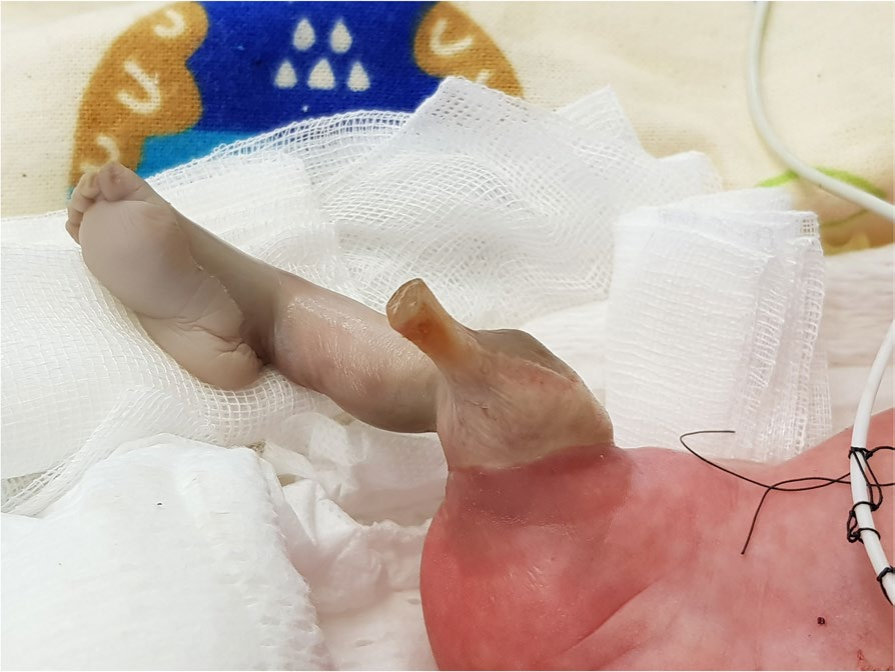
Right lower extremity ischemic necrosis at delivery

**Fig. 2:**
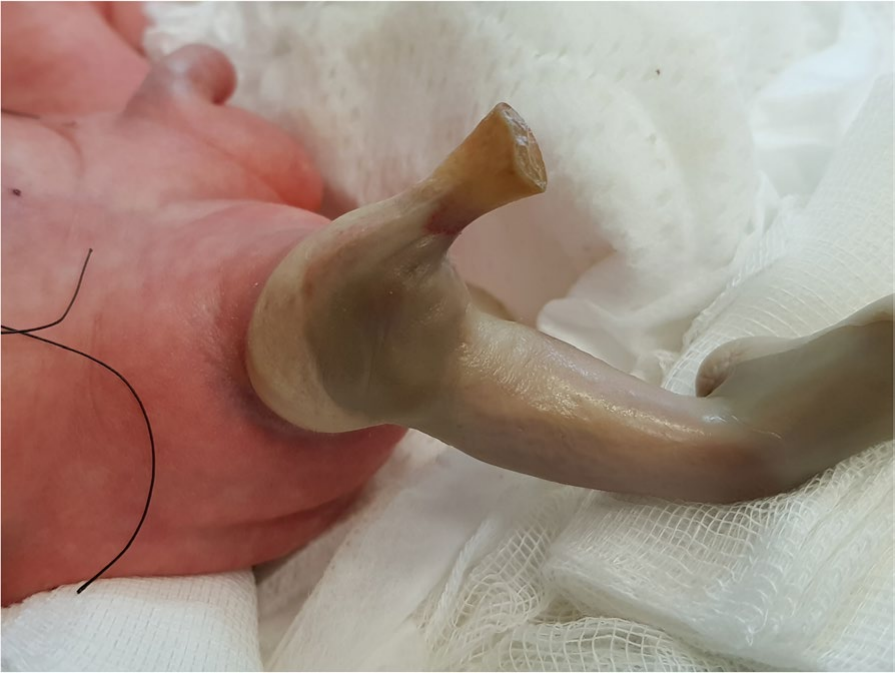
Right lower extremity ischemic necrosis at delivery

Twin I (the recipient twin) birth’s weight was 1340 g with an Apgar score of 7/8 at 1 and 5 min. Twin II (the donor twin) birth’s weight was 1240 g with an Apgar score of 5/8 at 1 and 5 min. The right lower extremity of the donor twin was noted as ischemic at the thigh level (shown in Fig. 1 and 2). Both newborns demanded intubation and mechanical ventilation with surfactant treatment due to respiratory distress.

The recipient twin had a recorded haemoglobin of 15.8 g/dL. The donor twin had a recorded haemoglobin of 11.8 g/dL. Therefore, postnatally there was no evidence of twin anemia/polycythemia sequence. Few hours after birth the impaired function of the donor’s right kidney was diagnosed. Unilateral renal ischemia on the same side as limb necrosis suggested the embolic cause of the complication. The donor twin’s right lower extremity was amputated at the Department of Paediatric Surgery 2 days after delivery. The twin had an uncomplicated recovery after procedure. Both twins had complications typical for preterm delivery but remained stable at the Neonatal Intensive Care Unit.

## Discussion and conclusions

Limb ischemic injury is a rare condition associated with twin-to-twin transfusion syndrome [12]. It is correlated with more conservative treatment including amnioreduction rather than successful SLP [14]. In most cases, vascular limb occlusion affects lower extremities (approximately 80%) of the recipient twins [15]. There are few theories suggesting the pathogenesis of the complication in recipient twins including polycythaemia/hyperviscosity syndrome, elevated level of vasoconstrictive hormones and venous thromboembolism [16].

In our case report, ischemic injury affected the donor twin lower extremity. The cause of the complication in donor twins is unknown. There are only a few cases described in the literature, but none of them explained the mechanism of in utero limb ischemia in donor twins [17]. Furthermore, most cases were related with advanced Quintero stage disease (in most of the reports stage III or IV) [14]. Therefore, the first hypothesis in our case (Quintero stage I; limb ischemia of the donor twin) was that the limb damage was related directly to SLP or due to the complication of the procedure- amniotic band disruption. After analysis of the case one of the thesis was disproved—SLP was stopped at the beginning of the procedure due to poor visibility. The second potential cause—amniotic band disruption was excluded due to the fact, that the fetuses where delivered via cesarean section in their amniotic sacs (En Caul Birth) with no evidence of the rupture of amniotic membranes. Based on that, it was suggested that the ischemic injury may be related to the main condition – TTTS. The coexisting impaired function of the right kidney and the length of the limb also suggested, that the ischemia of the lower extremity had occurred with no correlation with the invasive procedure, but as a result of thromboembolism.

In utero limb ischemia is a severe complication of TTTS. It occurs with the incidence rate of 0.5% [15]. Based on recent systematic review invasive procedures improve survival rates of twins in pregnancies complicated with TTTS Stage 1. Consequently, more cases are going to be qualified to SLP in early stages of TTTS [18]. Thus, neonates and obstetricians should be aware of the complication and before any treatment inform the patients about the risk of in utero limb injury in TTTS complicated twins. This case is unusual, because the ischemia of the lower extremity occurred in the donor twin in monochorionic twin pregnancy complicated with TTTS stage I. Therefore, it gives new insight into prenatal limb ischemic injury. Our case report supports the hypothesis that in utero limb ischemia is rather a complication of TTTS than SLP. However, further research is needed to reveal the mechanism of the ischemic limb injury in monochorionic twin pregnancies complicated with TTTS.

## Data Availability

All data generated or analysed during this study are included in this published article and its supplementary information files.

## Abbreviations

TTTS: Twin-to-twin transfusion syndrome
SLP: Selective laser photocoagulation
